# Brain white matter integrity in heroin addicts during methadone maintenance treatment is related to relapse propensity

**DOI:** 10.1002/brb3.436

**Published:** 2016-01-24

**Authors:** Wei Li, Jia Zhu, Qiang Li, Jianjun Ye, Jiajie Chen, Jierong Liu, Zhe Li, Yongbin Li, Xuejiao Yan, Yarong Wang, Wei Wang

**Affiliations:** ^1^ Department of RadiologyTangdu HospitalThe Fourth Military Medical UniversityXi'anShaanxiChina

**Keywords:** Diffusion tensor imaging, heroin addiction, methadone maintenance treatment, relapse propensity, white matter integrity

## Abstract

**Introduction:**

Cognitive deficits caused by heroin‐induced white matter (WM) impairments hinder addicts' engagement in and benefit from treatment. The predictive value of WM integrity in heroin addicts during methadone maintenance treatment (MMT) for future relapse is unclear.

**Methods:**

Forty‐eight MMT patients were given baseline diffusion tensor imaging scans and divided into heroin relapsers (HR, 25 cases) and abstainers (HA, 23 cases) according to the results of 6‐month follow‐up. Intergroup comparisons were performed for fractional anisotropy (FA), radial diffusivity (RD), and axial diffusivity (AD). The correlation between diffusion tensor imaging indices and the degree of heroin relapse was analyzed.

**Results:**

Compared with HA group, HR group had reduced FA in the right retrolenticular part, left anterior and posterior limb of internal capsule, bilateral anterior corona radiata, and right external capsule. Three out of the six regions showed increased RD, with no changes in AD. The FA and AD values in the left posterior limb of internal capsule correlated negatively with the heroin‐positive urinalysis rate within follow‐up.

**Conclusions:**

Lower WM integrity in MMT patients may add to neurobiological factors associated with relapse to heroin use. Strategies for improving WM integrity provide a new perspective to prevent future relapse to heroin abuse.

## Introduction

Heroin addiction is an illness characterized by its chronic and relapsing nature. Despite a variety of effective therapies for heroin addiction (Preston et al. 2000), the relapse rate remains high (Tang et al. 2006). Monitoring of therapeutic outcome and development of new therapeutic interventions for drug addiction become crucial (Volkow and Baler 2013). Neurocognitive factors such as inhibitory control, reward processing, working memory, decision making, and overall cognitive impairment play an important role in the risk of relapse in most substance addictions (Kosten 1986; Allsop et al. 2000; Grusser et al. 2004; Brewer et al. 2008; Streeter et al. 2008; Xu et al. 2010; Sorg et al. 2012). However, hidden neurobiological factors that contribute to the risk of relapse after treatment for addictions have not been well understood. More objective and quantitative methods are required to predict the risk of relapse.

Noninvasive imaging techniques provide biomarkers such as the blood flow, morphology, and metabolites of the brain, which have been found to be related to substance addiction and relapse after treatment. Lower frontal cerebral baseline blood flow has been found in relapsers compared with those who remained abstinent from alcohol abuse (Noel et al. 2002). In a morphological study, alcohol relapsers had a smaller total surface area in the brain reward system and lesser volumes in the right middle frontal gyri and bilateral orbitofrontal cortex than abstainers (Durazzo et al. 2011). Additionally, the tissue volumes in bilateral orbitofrontal cortex and surrounding white matter (WM) were smaller in future relapsers than in future abstainers at entry into alcohol dependence treatment (Cardenas et al. 2011). A diffusion tensor imaging (DTI) study demonstrated that better WM integrity in extensive brain regions at treatment onset was associated with longer abstinence of cocaine‐dependent individuals (Xu et al. 2010), suggesting the prognostic value of WM integrity for future relapse. Moreover, a number of factors have been shown to be predictive of treatment outcome in different substance addictions, such as lower baseline metabolite levels in the frontal and temporal lobe, greater activation of the striatum, anterior cingulate and medial prefrontal cortex under drug cues, and functional alteration of cognitive control‐related brain regions (Grusser et al. 2004; Brewer et al. 2008; Durazzo et al. 2008, 2010). Although studies show the correlation between brain alterations and future relapse propensity in addicts, the relationship between WM integrity in heroin addicts under methadone maintenance treatment (MMT) and the treatment outcome has not been fully investigated.

Prevention of relapse is of great importance in the treatment of addiction. A better understanding of relapse‐related functional and structural brain alterations may contribute to the design of effective, individualized treatment strategies. DTI has been widely used to examine and quantify WM integrity in terms of microstructure and physiological states (Lim and Helpern 2002). Several DTI indices can be used to quantitatively measure the physiological states of WM (Lim and Helpern 2002). Fractional anisotropy (FA) reflects the Brownian motion of water molecules constrained by the brain tissue, while axial diffusivity (AD) and radial diffusivity (RD) are useful in reflecting potential pathological changes in WM (Deo et al. 2006; Hasan and Narayana 2006; Herrera et al. 2008). Song and coworkers demonstrated that a significant decrease in AD with a sight, but not significant change in RD is representative of axonal damage, while an increase in RD with no change in AD is specifically suggestive of demyelination (Song et al. 2002, 2005).

This study aimed to explore the neurobiological factors during MMT associated with heroin relapse by DTI analysis of WM integrity. We examined whether brain WM integrity differed between addicts who relapsed and those who maintained abstinence from heroin abuse during a 6‐month follow‐up. The hypothesis was that the baseline FA and AD of individuals with relapse would be lower while the baseline RD would be higher than those in abstainers, due to axon compromise and demyelination, respectively. To confirm the relationship between baseline DTI indices and future relapse, we introduced the degree of relapse and analyzed its correlation with DTI indices. The results are expected to provide an insight into the strategies for reducing relapse to heroin addiction.

## Method

### Participants

Sixty‐nine male former chronic heroin addicts undergoing MMT were recruited from the outpatients of the Baqiao Methadone Maintenance Treatment Center in Xi'an, Shaanxi Province, China. All subjects were smokers. Inclusion criteria included: (1) meeting the diagnostic criteria of DSM‐IV (Diagnostic and Statistical Manual of Mental Disorders, Fourth Edition) for heroin addiction; (2) volunteering to receive long‐term and stable treatment at the MMT center; (3) taking MMT for at least 3 months and receiving a stable dose for at least 1 month before baseline; and (4) being strongly right handed as judged by the Edinburgh Handedness Inventory (Oldfield 1971). The exclusion criteria were: (1) neurological signs and/or a history of neurological disease; (2) a current medical illness; (3) addiction to any psychoactive substance other than heroin; (4) daily consumption of alcohol; (5) a history of head trauma; (6) a cardiovascular, pulmonary or systemic disease; (7) a fear of confined spaces; and (8) any contraindication to magnetic resonance imaging (MRI) examination. The potential impact of the most common comorbid psychiatric symptoms including anxiety and depression was evaluated using the Beck Depression Inventory II (BDI‐II) (Beck et al. 1996) and Hamilton Anxiety Scale (HAMA) (Hamilton 1959), respectively.

This study was approved by the Institutional Board of the Fourth Military Medical University, Xi'an, China. As required by the Institutional Review Board of the Tangdu Hospital, all subjects were informed of the experimental details and aims of the study, and written consent for their involvement was collected from each subject.

### Longitudinal clinical follow‐up

Methadone maintenance treatment was performed on each subject for at least 3 months before baseline and throughout the whole period of follow‐up after MRI scanning. The baseline for investigation was defined as the time point of MRI scanning. A 6‐month follow‐up was started at baseline by performing a structured interview along with urine test monthly to gather information on heroin intake and MMT status, similar as the design used previously (Sorg et al. 2012; Li et al. 2014). The focus was to determine whether the subjects relapsed to heroin abuse or temporarily withdrew from heroin abuse in the follow‐up period. Relapse to heroin use was assessed at a fixed time point in every month by the follow‐up interview and urine test. Participants were considered as relapsers if the urine test was positive or a self‐reported heroin use was received at any time during the follow‐up period.

### Image acquisition

All MRI scanning was performed at baseline using a 3.0T GE Signa Excite HD whole‐body MRI system, with a gradient strength of 40 mT/m, a slew rate of 150 T/m/sec, and an eight‐channel head coil (GE Medical Systems, Milwaukee, WI, USA). Routine T_2_‐weighted imaging sequences were used to exclude gross cerebral abnormality by an experienced radiologist. DTI data were collected using a spin‐echo echo‐planar imaging sequence and full brain axial sections were acquired with the following parameters: number of gradient orientations = 25, matrix = 128 × 128, field of view = 24 cm × 24 cm, repetition time = 7600 ms, echo time = 61.5 ms, number of excitations = 2, slice thickness = 4 mm without gap, and *b* value = 1000 s/mm^2^. The whole scanning protocol for each individual took about 10 min.

### DTI data processing

Diffusion Toolbox (FDT) and Tract‐Based Spatial Statistics (TBSS) from FMRIB's Software Library (FSL, http://www.fmrib.ox.ac.uk/fsl/) were used for preprocessing and WM microstructure analysis (Smith et al. 2006). Eddy current distortion and head motion in raw datasets were corrected using FDT followed by removal of background noise and nonbrain tissues using Brain Extraction Tool. The parameter maps including FA, RD (RD = [*λ*_2_ + *λ*_3_]/2) and AD (AD = *λ*_1_) were calculated using DTIFIT from FSL (Lim and Helpern 2002).

A standard TBSS approach was employed for whole‐brain voxel‐wise between‐group statistical comparisons. The FA image from each subject was nonlinearly transformed to the FMRIB58 FA template in Montreal Neurological Institute (MNI) space. Then, the dimension of the image was resampled at a resolution of 1 × 1 × 1 mm^3^. A mean FA map was generated based on the registered FA images from all subjects and skeletonized to outline the centers of all fiber bundles. In this step, the threshold of the mean skeleton image was set at the FA value of 0.2. Then, the registered FA map of each subject was transformed onto the group mean skeleton by filling with the FA values from the nearest relevant tract center (Lin et al. 2012). RD and AD maps underwent the same transformation using the algorithm defined by the matrix generated from FA map transformation. The obtained data were prepared for whole‐brain voxel‐wise statistical analysis across subjects between HR and HA groups.

### Statistical analysis

#### Voxel‐wise analysis for FA and ROI‐wise analysis for AD and RD between groups

The FA values were compared between two groups and the skeletonized FA data were fed into voxel‐wise statistical analysis, based on a nonparametric approach utilizing permutation test theory by a randomized tool in FSL (http://www.fmrib.ox.ac.uk/fsl/randomise/index.html). The number of permutation tests was set to 5000 (Nichols and Holmes 2002; Qiu et al. 2013). Age, duration of smoking, and history of methadone used before baseline showed intergroup differences; therefore, these variables were included as covariates to ensure that any observed difference of FA between groups was independent. Threshold‐free cluster enhancement (TFCE) was used to identify clusters with significant between‐group differences, and family‐wise error (FWE) with a significant threshold of *P *<* *0.05 was used for multiple comparison correction. The most probable anatomic localization of each significant cluster was determined by the AtlasQuery tool of FSL, Johns Hopkins University (JHU)‐ICBM‐DTI‐81 WM labels atlas and JHU WM tractography atlas in MNI space (Qiu et al. 2013). Clusters showing a significant difference between groups were saved as cluster masks. To explore the microstructural mechanisms of the observed FA changes, the mean RD and AD values of the regions of interest (ROIs) constrained by the cluster masks from each subject were extracted using fslmeants in FSL (http://fsl.fmrib.ox.ac.uk/fsl/fslwiki/Fslutils). Intergroup differences in RD and AD values in these ROIs were calculated with a two‐sample t‐test using a statistical significance level of *P *<* *0.05.

#### Comparison of demographic data between groups

Between‐group differences in demography characteristics, including age, years of education, history of smoking, duration and dose of methadone use before/after baseline, and heroin consumption before baseline, were tested using a two‐sample *t*‐test.

#### Correlation analysis

Spearman correlation analysis was used to identify the correlation between DTI indices (mean FA, RD, and AD values in the ROIs) and the positive urinalysis rate of HR within the 6‐month follow‐up period after baseline. The positive urinalysis rate was calculated by the proportion of positive urinalyses in all urinalyses of every patient in HR group. The general linear model was used to regress out the covariates of nuisance including age, years of education, duration and dosage of smoking, and heroin/methadone use. Due to the intergroup difference of the age, duration of smoking and history of methadone used prebaseline, we checked the potential correlations between these variates and the states of heroin relapse (i.e., relapse or abstinence) or the DTI indices, using the binary logistic regression and pearson correlation analysis, respectively. Two‐sample t‐test and correlation analysis were performed using the Statistical Package for Social Sciences version 13.0 (SPSS Inc., Chicago, IL, USA). A *P‐*value of 0.05 or less was considered statistically significant.

## Results

### Demographic and clinical characteristics

Of the 69 participants who underwent baseline DTI scans, 21 were excluded from the analysis because of incomplete brain coverage, head movement, gross cerebral abnormality, and incomplete follow‐up. According to the defined criteria for relapse, the rest 48 subjects were divided into two groups: 25 for HR and 23 for HA. All the heroin addicts in this study used heroin by snorting and 19 of them involved syringe injection.

No significant difference was obtained in education level, number of cigarettes smoked per day, previous heroin consumption history, or daily methadone maintenance dosage between HR and HA groups (Table 1). The mean age of HA group was significantly greater than that of HR group. HA group showed a significantly longer duration of MMT, a longer history of cigarette smoking, and a higher accumulated dosage of methadone used previously than HR group.

**Table 1:** Demographic and clinical characteristics of 48 subjects (mean ± SD)

Characteristics	HA (*N* = 23)	HR (*N* = 25)	Test‐value (*t*)	*P*‐value
Age (years)	39.4 ± 7.7	32.3 ± 6.6	3.45	0.001^a^
Education level (years)	8.8 ± 2.4	9.7 ± 2.2	−1.32	0.20
Number of cigarettes smoked (per day)	18.7 ± 9.2	19.8 ± 10.4	−0.40	0.69
Duration of cigarette smoking (years)	21.7 ± 8.8	15.4 ± 7.4	2.70	0.01^a^
Accumulated dosage of heroin abuse (g)	946.6 ± 1233.9	976.7 ± 1474.0	−0.08	0.94
Duration of heroin abuse (months)	101.6 ± 80.6	72.8 ± 72.9	1.30	0.20
Prebaseline accumulated dosage of MMT (g)	42.7 ± 35.3	24.0 ± 15.6	2.35	0.03^a^
Prebaseline duration of MMT (months)	30.6 ± 19.3	19.1 ± 11.4	2.49	0.02^a^
Daily methadone dosage (ml/day)	45.7 ± 17.3	41.8 ± 14.1	0.85	0.40
Beck Depression Inventory score	10.7 ± 8.8	9.6 ± 9.0	0.43	0.67
Hamilton Anxiety Scale score	10.0 ± 12.4	8.0 ± 7.6	0.71	0.48

HA, heroin abstainers; HR, heroin relapsers; MMT, methadone maintenance treatment.

a Significant difference, *P *<* *0.05.

### TBSS results of FA

**Figure 1:**
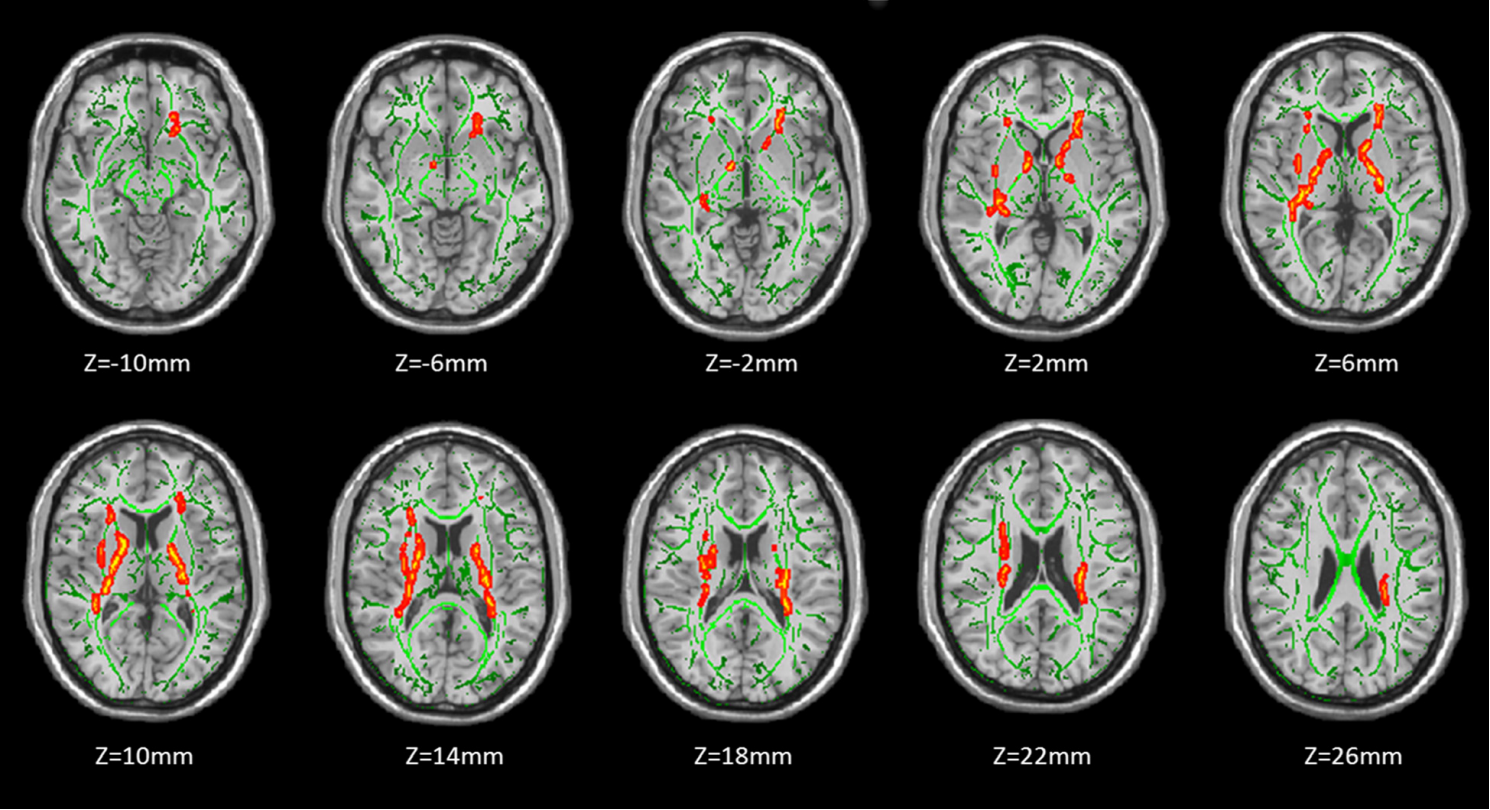
TBSS analysis of fractional anisotropy (FA) between heroin relapsers (HR) and abstainers (HA). Red areas indicate where FA was significantly lower in HR group than in HA group (*P *<* *0.05, corrected by TFCE and FWE). To help visualization, regions with reduced FA (red) were thickened using the ‘tbss_fill’ script implemented in FSL. Green color shows ‘group mean_FA_skeleton’ and the number below each brain image indicates *Z* coordinate in MNI space. The left side of the image corresponds to the right hemisphere of the brain.

Fractional anisotropy was significantly lower in HR group than in HA group in the right retrolenticular part of internal capsule, left posterior limb of internal capsule, bilateral anterior corona radiata, left anterior limb of internal capsule, and right external capsule (*P *<* *0.05, corrected by TFCE and FWE) (Fig.**** 1, Table 2). No WM regions showed significant reduction of FA in HA group compared with HR group.

**Table 2:** White matter regions with reduced fractional anisotropy in heroin relapsers compared with abstainers

Location	Voxel size	R/L	MNI (mm)			*P*‐value
			*X*	*Y*	*Z*	
Retrolenticular part of internal capsule	1293	R	27	−22	8	0.039^a^
Posterior limb of internal capsule	850	L	−25	−16	6	0.046^a^
Anterior corona radiata	103	R	25	22	10	0.048^a^
Anterior corona radiata	368	L	−23	28	2	0.046^a^
Anterior limb of internal capsule	37	L	−16	16	−2	0.049^a^
External capsule	7	R	28	10	16	0.049^a^

MNI, Montreal Neurological Institute; R, right; L, left.

*X*,* Y*,* Z*: the location of peak value in the cluster (MNI coordinates).

a Significant difference, *P *<* *0.05, TFCE and FWE corrected.

### Intergroup differences of RD and AD

The mean RD and AD values extracted from the six regions with significantly reduced FA were further analyzed (Table 3). Results showed that RD was significantly increased in three out of the six regions (*P *<* *0.05), including the right retrolenticular part of internal capsule, left posterior limb of internal capsule, and right external capsule. No significant changes in AD were observed between groups (*P *>* *0.05).

**Table 3:** Comparison of mean RD and AD in white matter regions with significant intergroup difference in FA

Location	R/L	RD			AD		
		HA	HR	*P*‐value	HA	HR	*P*‐value
Retrolenticular part of internal capsule	R	0.46 ± 0.03	0.48 ± 0.02	0.001^a^	1.28 ± 0.03	1.28 ± 0.02	0.95
Posterior limb of internal capsule	L	0.41 ± 0.03	0.43 ± 0.03	0.017^a^	1.38 ± 0.04	1.37 ± 0.03	0.26
Anterior corona radiata	R	0.52 ± 0.04	0.54 ± 0.03	0.091	1.26 ± 0.07	1.23 ± 0.06	0.23
Anterior corona radiata	L	0.56 ± 0.04	0.58 ± 0.03	0.161	1.30 ± 0.06	1.28 ± 0.05	0.11
Anterior limb of the internal capsule	L	0.51 ± 0.04	0.52 ± 0.04	0.300	1.29 ± 0.08	1.27 ± 0.08	0.48
External capsule	R	0.55 ± 0.04	0.58 ± 0.04	0.018^a^	0.95 ± 0.06	0.94 ± 0.05	0.59

RD, radial diffusivity; AD, axial diffusivity; FA, fractional anisotropy; R, right; L, left; HA, heroin abstainer; HR, heroin relapsers.

a Significant different, *P *<* *0.05.

RD and AD: ×10^−3^ mm^2^/s.

### Correlation between DTI indices and degree of heroin relapse

**Figure 2:**
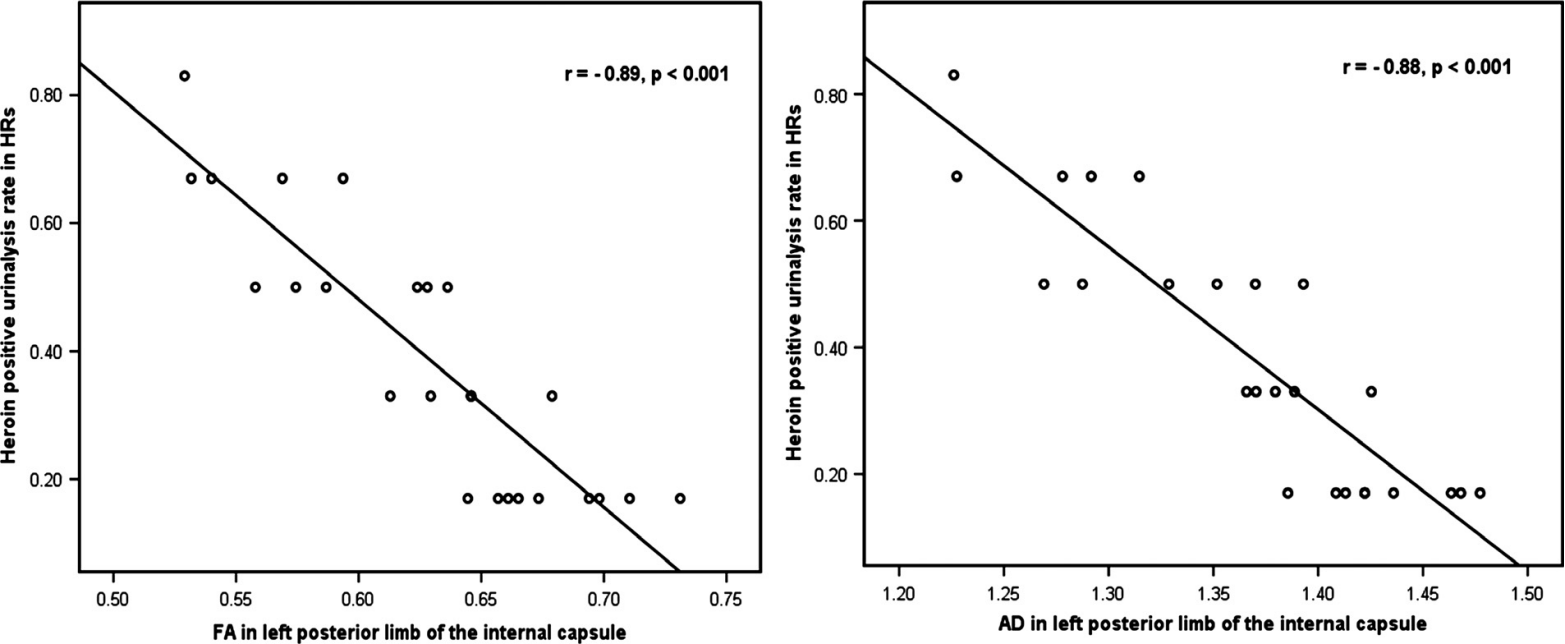
Graphical presentation of significant correlations between the heroin‐positive urinalysis rate (corrected for age, years of education, duration and dosage of smoking, and heroin/methadone use) within 6‐month follow‐up after baseline and FA/AD values in the left posterior limb of internal capsule in heroin relapsers.

ROI‐based correlation analysis demonstrated that in HR group, the FA and AD values in the left posterior limb of internal capsule significantly negatively correlated with the heroin‐positive urinalysis rate within 6‐month follow‐up (*r* = −0.89, *P *<* *0.001; *r* = −0.88, *P *<* *0.001, survived after Bonferroni correction) (Fig.**** 2). No significant correlation between DTI indices and the heroin‐positive urinalysis rate was found in other ROIs. The logistic regression results showed that all of variates which showed significant intergroup difference had no significant effects on group membership. While we found that age were negatively correlated with the FA and AD values in anterior corona radiata (*r* = −0.44, *P *=* *0.028 in right and *r* = −0.42, *P *=* *0.037 in left) and in internal capsule (*r* = −0.43, *P *=* *0.033 in left posterior limb of internal capsule and *r* = −0.35, *P *=* *0.006 in left anterior limb of internal capsule). Duration of cigarette smoking were negatively correlated with the AD values in right retrolenticular part of internal capsule and left anterior limb of internal capsule (*r* = −0.33, *P *=* *0.024 and *r* = −0.39, *P *=* *0.006, respectively).

## Discussion

This study compared baseline WM integrity during MMT between heroin addicts who relapsed (HR) and those who did not (HA) within 6‐month follow‐up. Significantly lower baseline FA was detected in HR group than in HA group in six WM regions. This result supports the hypothesis that lower baseline WM integrity during MMT could predict the heroin relapse. Our observation of significantly increased RD in three regions with reduced FA also supports the above hypothesis. In HR group, FA and AD values in the left posterior limb of internal capsule correlated negatively with the heroin‐positive urinalysis rate. This finding may highlight the predictive value of this WM region for the severity of heroin relapse.

Decreased WM integrity in the relapsers was found mainly in the internal/external capsule and the anterior corona radiata. These regions are regarded as important components of the neural circuit involved in substance addiction. As part of the limbic–thalamo–cortical circuitry, anterior corona radiata involves thalamic projections from the internal capsule to the prefrontal cortex areas, which are critical in reward seeking and inhibitory control in substance dependence and relapse (Kalivas and Volkow 2005; Feltenstein and See 2013). Disinhibition resulted from frontal WM damage may cause additional behavioral changes, owing to the impact of immediate reward saliency and prepotent response tendencies (Robbins 1996; Sorg et al. 2012). Previous studies reported that opioid‐dependent subjects showed significantly decreased FA in the internal and external capsules (Upadhyay et al. 2010). Our findings have extended WM impairment in the internal capsule, which may contribute to the disconnected function of prefrontal cortex and thalamus/striatal, parietal, temporal, and occipital cortices (Zarei et al. 2007; Zhang et al. 2010). Although there was no direct relationship between cognitive deficits and WM integrity abnormalities identified in this study, previous work has confirmed WM impairments underlying abbreviate cognitive function and behavior disturbances (Moeller et al. 2005; Chung et al. 2007; Harris et al. 2008; Bell et al. 2011; Vargas et al. 2014). Therefore, the neurobiological abnormalities of identified regions in our study may be related to cognitive, emotional, and behavioral disturbances that confer risk for the relapse/remit cycle common in substance‐use disorders (Kalivas and Volkow 2005; Kalivas and O'Brien 2008; Crews and Boettiger 2009). Additionally, the FA and AD values in the left posterior limb of internal capsule were in significant negative correlations with the positive urinalysis rate of HR group. Presumably, lower WM integrity in this region of relapsers betokens higher dependence on heroin abuse as well as higher severity of relapse.

Although still under debate, RD is generally believed to mainly reflect the integrity and thickness of myelin sheets, while AD reflects the integrity of axons and the organization of fiber structure (Song et al. 2002, 2003). Results from the proposed study suggest that the compromised WM integrity in individuals at baseline reflects the injury of myelin, which shows an increased risk of relapse to heroin abuse under MMT. It has been confirmed that WM integrity is reversible by physical treatments (e.g., melodic intonation therapy to enhance WM integrity in aphasic patients) or pharmacological treatments (e.g., triiodothyronine to improve WM integrity and behavioral symptoms of mice with demyelination) (Harsan et al. 2008; Schlaug et al. 2009). Therefore, altered WM integrity found in HR is possible to serve as a new biomarker and provide a considerable therapeutic target. Further investigation is required to determine whether improving the integrity of myelin in heroin addicts could help to reduce the risk of heroin use during MMT.

Age and the status of cigarette smoking should be considered in interpreting the relationship between WM integrity and heroin relapse under MMT. Age‐related declines in cerebral WM integrity have been well documented (Minati et al. 2007; Carmichael and Lockhart 2012; Madden et al. 2012; Bennett and Madden 2013). According to the literature, older adults have decreased FA and increased RD relative to younger adults (Zahr et al. 2009; Bennett et al. 2010; Charlton et al. 2010; Teipel et al. 2010; Burgmans et al. 2011), the significant negative correlations between age and the FA and AD values in this study were consistent with the previous results. In this study, the subjects of HR group were younger than those of HA group, but the former had lower WM integrity than the latter. Also, the relapse of heroin addiction was reported to be unassociated with age (Termorshuizen et al. 2005). Therefore, we conclude that age is not a significant factor responsible for alteration of WM integrity observed in HR. The relationship between smoking and WM integrity has been investigated using DTI, but the published results are inconsistent. Studies generally accept the model of higher FA with cigarette exposure during adolescence and declined FA with continued smoking in adulthood (Paul et al. 2008; Gons et al. 2011; Hudkins et al. 2012). A recent study has reported a significant positive correlation between reduction of WM integrity and duration of smoking (Lin et al. 2012), which was also validated by the negative correlation between the AD values and the duration of smoking in this study. Therefore, the intergroup difference in smoking duration may not account for the WM impairment of HR at baseline.

There were several limitations in this study. First, the accumulated dosage and duration of MMT prebaseline were unmatched. Previous work found a correlation between high methadone dosage (i.e., >80 mg/day) and lower risk of relapse among those who were stable in MMT program; however, the magnitude of the effect of methadone dose was considered to be only moderate, and those using methadone in high dosage were still at a considerable risk of relapse (Termorshuizen et al. 2005). To the best of our knowledge, the specific impact of MMT duration on relapse remains indefinite. Stimmel et al. (1987) observed that the risk of relapse increased 20% for each additional year in the duration of treatment. Nevertheless, we admit that the unmatched prebaseline MMT status is a potential influential factor of MMT outcome. Therefore, the status of prebaseline MMT was used as the control variable for intergroup comparison to diminish their interference to the results. Second, the prebaseline relapse rate of the MMT patients was not included in this study. Patients who did not relapse within 6‐months may relapse to heroin abuse in the past or in a longer follow‐up period. Therefore, the presented results may just show a tendency of heroin relapse‐related WM alterations. Third, to test heroin relapse by fixed monthly urine screening could be problematic. Although the patients cooperated well, all of them were outpatients and there was no guarantee for the accuracy of their heroin usage. Additionally, we only investigated male subjects, while a possible gender difference in relapse susceptibility was unexplored. Thus, the current findings may not be generalized to all individuals, it is worth of further investigation including female addicts and exploring potential gender‐related susceptibility in the relationship between WM integrity and risk of heroin relapse.

In summary, this study demonstrated that future relapsers have lower WM integrity than abstainers under MMT. Since lower WM integrity is associated with higher frequency of relapse, it may add to neurobiological factors associated with relapse to heroin use. Furthermore, our findings provide insight into the strategies for reducing relapse risk to heroin addiction by improving WM integrity, which requires further test.

## Conflict of Interest

The authors have no conflicts of interest to declare.
